# Acrylic Acid Plasma Coated 3D Scaffolds for Cartilage tissue engineering applications

**DOI:** 10.1038/s41598-018-22301-0

**Published:** 2018-03-01

**Authors:** Pieter Cools, Carlos Mota, Ivan Lorenzo-Moldero, Rouba Ghobeira, Nathalie De Geyter, Lorenzo Moroni, Rino Morent

**Affiliations:** 1 Research Unit Plasma Technology, Department of Applied Physics, Sint-Pietersnieuwstraat 41 B4, Ghent University, 9000 Ghent, Belgium; 2 Department of Complex Tissue Regeneration, MERLN Institute for Technology Inspired Regenerative Medicine, Universiteitssingel 40, University of Maastricht, 6200 MD Maastricht, The Netherlands

## Abstract

The current generation of tissue engineered additive manufactured scaffolds for cartilage repair shows high potential for growing adult cartilage tissue. This study proposes two surface modification strategies based on non-thermal plasma technology for the modification of poly(ethylene oxide terephthalate/poly(butylene terephthalate) additive manufactured scaffolds to enhance their cell-material interactions. The first, plasma activation in a helium discharge, introduced non-specific polar functionalities. In the second approach, a carboxylic acid plasma polymer coating, using acrylic acid as precursor, was deposited throughout the scaffolds. Both surface modifications were characterized by significant changes in wettability, linked to the incorporation of new oxygen-containing functional groups. Their capacity for chondrogenesis was studied using ATDC5 chondroblasts as a model cell-line. The results demonstrate that the carboxylic acid-rich plasma coating had a positive effect on the generation of the glucoaminoglycans (GAG) matrix and stimulated the migration of cells throughout the scaffold. He plasma activation stimulated the formation of GAGs but did not stimulate the migration of chondroblasts throughout the scaffolds. Both plasma treatments spurred chondrogenesis by favoring GAG deposition. This leads to the overall conclusion that acrylic acid based plasma coatings exhibit potential as a surface modification technique for cartilage tissue engineering applications.

## Introduction

Articular cartilage is the connective tissue that allows for the frictionless movement of bone within the synovial joints of the body. Its extracellular matrix consists of a combination of different types of collagen and proteoglycans that together are responsible for the viscoelastic and swelling properties of the tissue. Unlike bone, articular cartilage lacks vascularization, a neural network and a lymphatic system^1,2^. Avascularity combined with a slow mitotic cycle of the matrix-encapsulated cells inherently limits its endogeneous capacity for cartilage tissue regeneration^3–5^. At present, there are no long-term solutions available for cartilage related injuries, which has a significant clinical and economic impact worldwide^6^.

*In vitro* tissue engineering approaches for cartilage repair have been extensively studied in the last decades using a multitude of engineering strategies^1,3–11^. Both natural and synthetic polymers have received considerable interest as substrate materials for the fabrication of 3D support structures. While natural source compounds, such as gelatin and alginates, are more bioactive and truly biodegradable, synthetic polymers are more predictable, reproducible, and scalable in terms of chemical and physical properties and offer a stronger structural support. Depending on the chosen scaffold fabrication technique and the end-application, both types of resources can be valid options. Among the different synthetic polymers, biodegradable thermoplastics such as Poly-L-lactic acid (PLLA), poly-ε-caprolactone (PCL) and poly (ethylene oxide terephthalate)/poly (butylene terephthalate) (PEOT/PBT) are of particular interest, as they are relatively cheap, commercially available, easily manipulated and exhibit excellent structural properties. Downsides are fixed degradation rates, acidic degradation products, low elasticity and limited bioactivity^12–15^. Most of these issues have been resolved over the last 2 decades via the synthesis of biodegradable copolymers that allow for the fine-tuning of the degradation rate, resulting in a more controlled formation of acidic degradation products. The elasticity limitations have been addressed by combining the aforementioned synthesis with proper additive manufacturing parameters^16–19^. Processing the materials into proper 3D support structures for cell growth can be done through a number of fabrication techniques, each with their own specific set of advantages and disadvantages^20–22^. Among those approaches, additive manufacturing of thermoplastic polymers is of great interest due to the fast production process, excellent pore interconnectivity and versatility in scaffold design and will therefore also be used in this work.

To extract a proper bioactive response from these types of 3D scaffolds is, however, still one of the main challenges today. Extensive literature is available on the enhancement of thermoplastic elastomers for cartilage repair in 2D configuration (films and flat surfaces) through surface modifications, yet literature on surface modifications of 3D scaffolds is rather limited, as the penetration of the micropores using wet-chemical approaches is challenging and a prolonged exposure to (harsh) chemicals often compromises the integrity of these delicate structures^23–26^. An alternative approach towards the effective modification of 3D polymeric scaffolds is non-thermal plasma technology (NTP). NTP is a well-established gas-based technique typically used for altering the surface chemical composition of any exposed substrate. When feeding an inert gas such as argon, air or helium to generate the plasma discharge, radical sites are generated, resulting in the incorporation of polar functional groups, a process that is often referred to as plasma activation. Besides the incorporation of functional groups, it is also possible to employ NTP to deposit thin polymer-like films onto the surface. When feeding the gaseous film precursor into the reactor after activation, but without employing the discharge, the process is referred to as plasma grafting. If a plasma discharge is active while feeding the precursor gas, the deposition process is defined as plasma polymerization. Unlike traditional polymerization reactions, plasma “polymers” are known to be extensively cross-linked, pinhole free, and highly adherent. Compared to wet-chemical deposition processes, plasma polymerization can be favorable for the deposition of thin films on geometrically complex biodegradable polymer structures, as it is 1) a solvent-free technique, thus generating no waste and avoids the use of toxic solvents, 2) time-efficient, with deposition runs typically no longer than 30 min, 3) gas-based, thus allowing for a more efficient penetration throughout the porous scaffold, 4) non-invasive, not altering the bulk properties of the used biodegradable polymer^24,27–30^.

In this comparative *in vitro* study, the cartilage regeneration capacity of ATDC5 chondroblasts seeded onto acrylic acid plasma coated and He plasma activated 3D printed scaffolds will be evaluated in 2 sets of experiments. The first experiment uses growth medium as such, without the addition of any soluble growth factors (no insulin, transferrin and selenium; ITS-free) to avoid possible interference of the growth factors with the modified scaffold surfaces, while the second experiment adds the ITS to enhance the chondroblast metabolic activity. Whereas changes in cell-material interactions for plasma treated and plasma grafted additive manufactured scaffolds have been explored before, literature on the full characterization of a plasma deposited coating throughout a printed scaffold and its effect on cell interactions is severely limited^31^. Moreover, to the best of our knowledge, the capacity for cartilage regeneration of plasma treated and acrylic acid plasma coated 3D scaffolds has not been described before. In this work, the well-established ATDC5 embryonal carcinoma-derived clonal cell-line will be used, as it is considered to be the standard model for studying chondrogenesis in the early stages of endochondral bone development^32^. We show that the 2 plasma treatments induce different cellular mechanisms to enhance chondrogenesis. PEOT/PBT will be used as additive manufacturing material, as it is a clinically relevant polymer that has been extensively studied *in vitro* and *in vivo*, exhibiting a slightly moderate wettability compared to more hydrophobic biodegradable polyesters such as PCL and PLLA^33–36^.

## Results and Discussion

### Physico-chemical analysis

**Figure 1:**
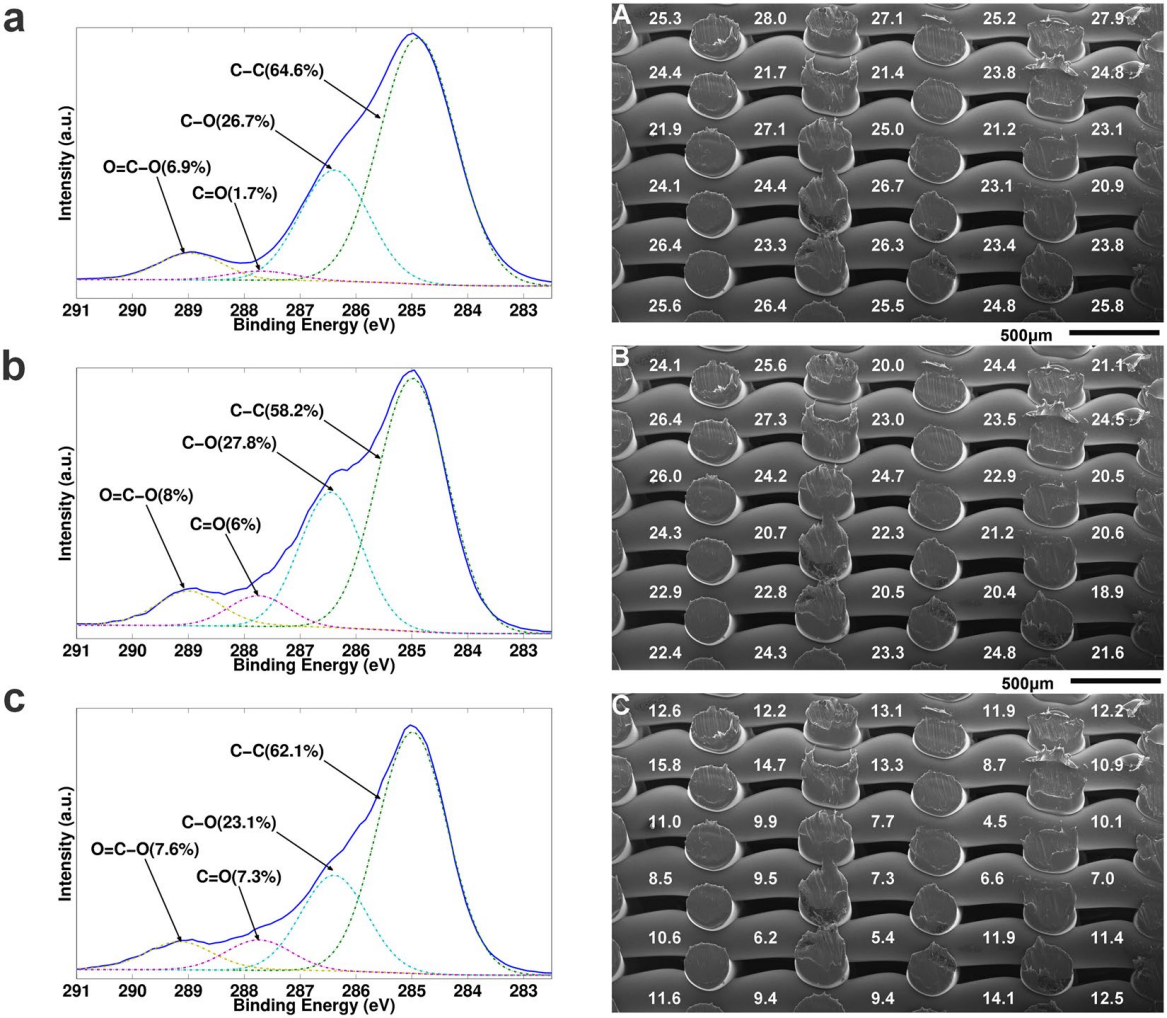
(**a**–**c**) XPS C1s deconvolution spectra for the untreated scaffold (**a**), the plasma activated scaffold (**b**) and the plasma coated scaffold (**c**). (**A**–**C**) XPS measurements throughout the porous scaffold: (**A**) O1s concentration of the He plasma activated sample; (**B**) O1s concentration of the acrylic acid plasma coated sample; (**C**) O=C-O concentration as determined from deconvolution of the C1s peak (peak intensity at 289.1 eV) of the plasma coated sample.

SEM analysis of the printed scaffold filaments showed a high level of consistency between the theoretically expected parameters (D1 = 250 µm, D2 = 650 µm, D3 = 170 µm) and the experimentally obtained results (D1 = 260 µm ± 20 µm, D2 = 655 ± 11 µm, D3 = 170 ± 7 µm) (Fig. 1C,D). Scaffolds showed 100% pore interconnectivity and were reproducible with the same characteristics each time they were fabricated. The total porosity of the scaffold was 55.6 ± 2.4%.

After exposing the scaffolds to the non-thermal He plasma activation and acrylic acid plasma polymerization steps, no visible microscopic changes were observed on the SEM micrographs. Analysis of the nano-topography using AFM (Table 1) revealed a native roughness of 24.9 ± 2.8 nm with a random orientation. After He plasma activation, the roughness non-significantly increased to a value of 27.0 ± 0.9 nm, showing that plasma activation had no obvious influence on the surface topography. After deposition of the acrylic acid coating, the roughness however significantly decreased to 18.3 nm ± 2.9 nm. A phenomenon which is typical for plasma deposition processes, as pits will fill faster compared to tops, something that was observed before^37^.

**Table 1:** physical properties of PEOT/PBT before and after plasma modification.

	Roughness AFM	WCA scaffold	WCA spin-coated	% O spin-coated
UNT	24.9 +/− 2.8 nm	125 ± 6°	61.0° +/− 1.9°	19.5 ± 1.8%
PAct	27.0 +/− 0.9 nm	0°	30.1 +/− 2.4°	25.9 ± 0.4%
PC	18.3 +/− 2.9 nm	0°	37.4 +/− 1.6°	23.3 ± 1.0%

WCA measurements were performed on the additive manufactured scaffolds to analyze the occurring changes in wettability (Table 1). The WCA changed from 125.4 ± 6° to 0° after He plasma activation and acrylic acid plasma coating, due to the capillary effect of the µ-pores, thus not qualifying as a true WCA value. Therefore, measurements were also performed on spin-coated poly (ethylene oxide)/poly (butylene terephthalate) samples to obtain a more nuanced view on the treatment efficiency (Table 1). For the untreated flat sample, a WCA of 61.0° ± 1.9° was found. After plasma activation, the contact angle decreased to 30.1 ± 2.4° while a slightly higher value of 37.4 ± 1.6° was obtained for the acrylic acid plasma coated samples. Measurements on both scaffolds as well as flat samples thus revealed a significant increase in hydrophilicity induced by the plasma treatments, while the flat samples indicated that the increase in wettability was slightly larger for the plasma activated samples. Combined with the information obtained from AFM measurements, it is clear that both plasma surface modifications are of a predominantly chemical nature. Compared to similar acrylic acid plasma coating processes, the observed WCA were found to be slightly higher. This could be assigned to the fact that in this study harsher deposition conditions were used to guarantee sufficient coating stability for long-term *in vitro studies*^38,39^.

XPS measurements were also performed on flat samples as well as throughout the scaffolds to quantify the chemical changes introduced by the He plasma activation and acrylic acid plasma polymerization step and their penetration efficiency throughout the scaffold. The flat samples (Table 1) show an increase in oxygen concentration from 19.5% to 25.9% after plasma activation. Upon acrylic acid plasma coating, the oxygen concentration again slightly decreases to 23.3%, data which can be directly correlated with the observed changes in WCA.

C1s deconvolution (Fig. 1b) shows very similar spectra for all 3 conditions with peaks at 285.0 eV (C-C), 286.4 eV (C-O), 287.4 (C=O) and 288.9–289.1 eV (O=C-O). Data analysis shows that after He plasma activation (Fig. 1b) an average increase of 4.3% for the peak at 287.4 eV (purple) can be noted, suggesting the efficient incorporation of the highly reactive aldehyde functional groups. This is in close agreement with literature on the modification of polyterephtalate surfaces with He non-thermal plasma^40^. The peak intensity of 288.9 eV (yellow), linked to esters is not significantly changing, but the FWHM does increase from 0.98 to 1.3, strongly indicating that the rigid chemical structure of PEOT/PBT copolymer at the surface is heavily perturbed. Based on literature, this suggests that besides the ester groups already present, a small percentage of carboxylic acid groups as well as peroxides are incorporated on the plasma activated sample^41^. For the plasma coated sample (Fig. 1c), an average increase of 5.6% of the peak at 287.4 eV (purple) is found, but more importantly, a shift of the peak at 288.9 eV to 289.1 eV (yellow) is observed. This shift strongly suggests that the majority of this peak’s intensity can be directly linked to the incorporation of carboxylic acid groups^42^. Based on the work done in previous studies, an estimate of about 40–45% of the O=C-O peak can be attributed to carboxylic acids. This would result in an incorporation of about 3–4% of carboxylic acid groups onto the flat surface^43^.

To analyze the induced changes throughout the interior, the scaffolds were cut in half and placed as such that the front of the scaffold is facing left and the back of scaffold facing right, the back being that part of the scaffold oriented towards the outer rim of the electrode. The results for the cross-sectional surface analysis of the scaffolds are presented in Fig. 1A–C. Surface analysis of the untreated scaffold revealed that the chemical surface composition did not change after the additive manufacturing step (oxygen content of 19.3% ↔ 19.5 ± 0.8%), indicating that the printing process does not alter the surface chemical composition. After the plasma activation step, a relatively homogeneous treatment is found throughout the scaffold, with a slight gradient in the middle of the scaffold and towards the back (right side of the scaffold image depicted in Fig. 1A. This is not abnormal, as those parts exposed to a lower He flow are subjected to a lower renewal rate of the active species, resulting in a marginally lower treatment efficiency. With an average value of 24.5%, the treatment efficiency situates itself slightly lower compared to the flat sample, but with a standard deviation of less than 2%, the surface treatment process can be considered as homogeneous. These results are in close relationship with the data of Jacobs *et al*. on the modification of PCL additive manufactured scaffolds with a He plasma at medium pressure, where variations up to 4% in treatment homogeneity were found^27^. Others found similar functional group distributions (1–4% gradient) when applying a low pressure plasma activation step for the modification of similar type of scaffolds^28,29^. After the acrylic acid plasma polymerization step (Fig. 1B), the changes between the top/front (left/top side of the image) and the center/back (bottom/right) of the scaffold are more pronounced. This was to be expected, as previous work on flat surfaces has shown that gradients in gas flow throughout the reactor, or in this case throughout the scaffold, have a pronounced effect on the surface chemical composition of the deposited coating^44^. Much the same as for the plasma activation, the regions in the center and facing the back of the gas flow suffer from a lower incorporation of oxygen (−8%) (Fig. 1B; minimum vs. maximum value recorded) and O-C=O functional groups (−10%) (Fig. 1C; minimum vs maximum value recorded) compared to the top/front of the scaffold. Compared to the flat samples, a significantly lower functional group incorporation efficiency was found. Again, this can be linked directly to a drop in the gas flux once it enters the scaffold, much the same as what was observed in earlier work^44^. Barry *et al*. also observed these steeper gradients and lower incorporation efficiency for the plasma coatings deposited throughout their scaffolds compared to plasma activated/grafted samples, as the lower reactor pressure did not compensate for the lower pore interconnectivity of their scaffolds^28^. However, the minimum amount of incorporated carboxylic acid groups is still around 2% (40–45% of 4.5%), which according to previous work is sufficient to significantly alter cell behavior^44^. Furthermore, Chen *et al*. showed that further increasing the COOH functional group density does not necessarily lead to an improved cell viability within the first 5 days of seeding^45^.

### Biological analysis

#### *In vitro* differentiation without biochemical induction

Four different scaffolds were submitted to *in vitro* investigation: i) a non-treated scaffold, acting as a negative control (UNT), ii) a non-treated scaffold with ITS added to the culture medium, acting as a positive control (ITS), iii) the plasma activated scaffold (PAct) and iv) the plasma coated scaffold (PC). The first series of experiments were conducted without the addition of ITS to study the performance of the surface modification without the influence of these external factors.

**Figure 2:**
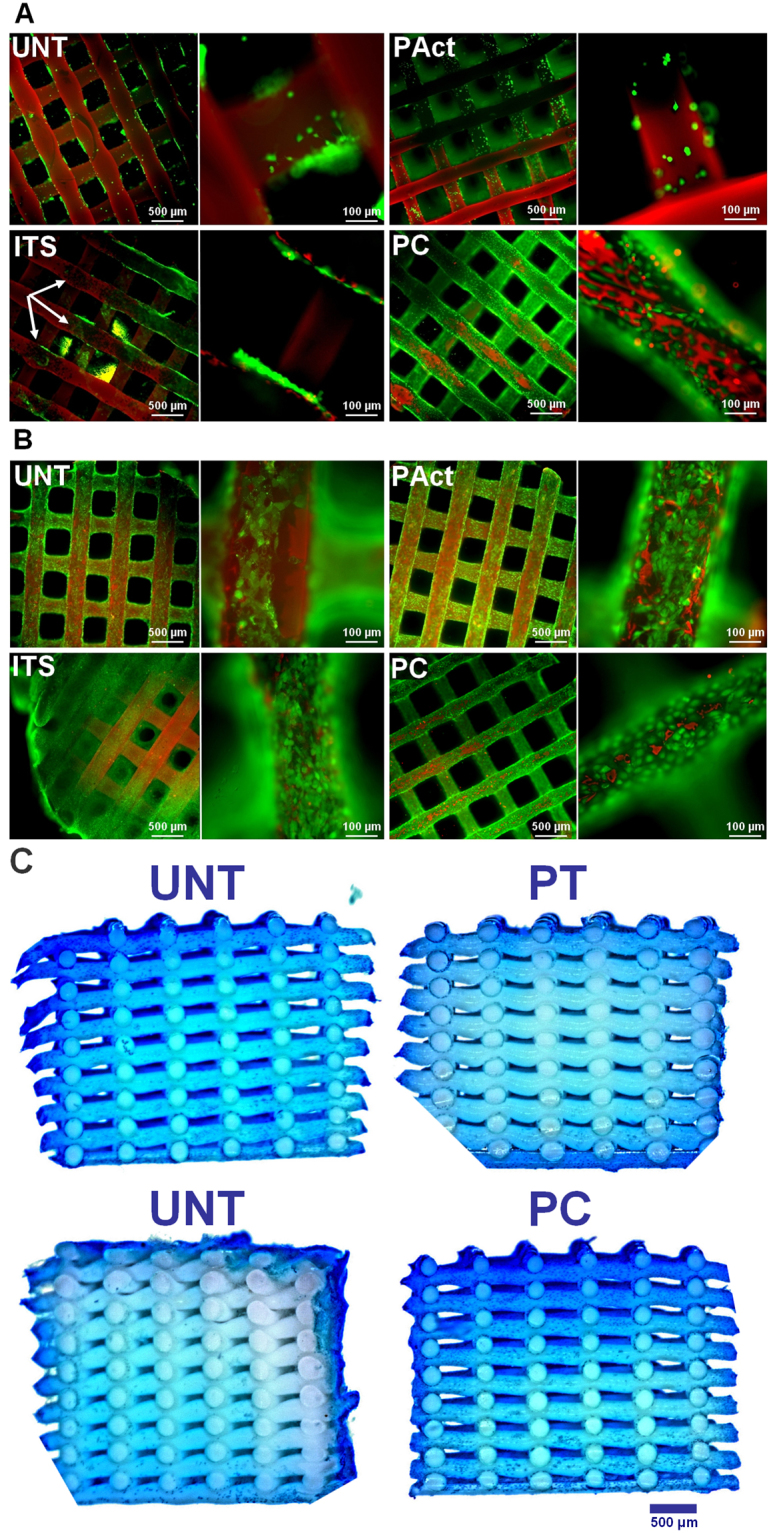
Fluorescent micrographs of live/dead stained samples (4x and 20x) of the first set of experiments at time points day 1 (**A**) and day 15 (**B**) for the untreated samples (UNT), plasma activated samples (PAct), positive control (ITS) and plasma coated samples (PC). Methylene blue stained cross-sections of the scaffolds for the first set of experiments at day 15 (**C**).

**Figure 3:**
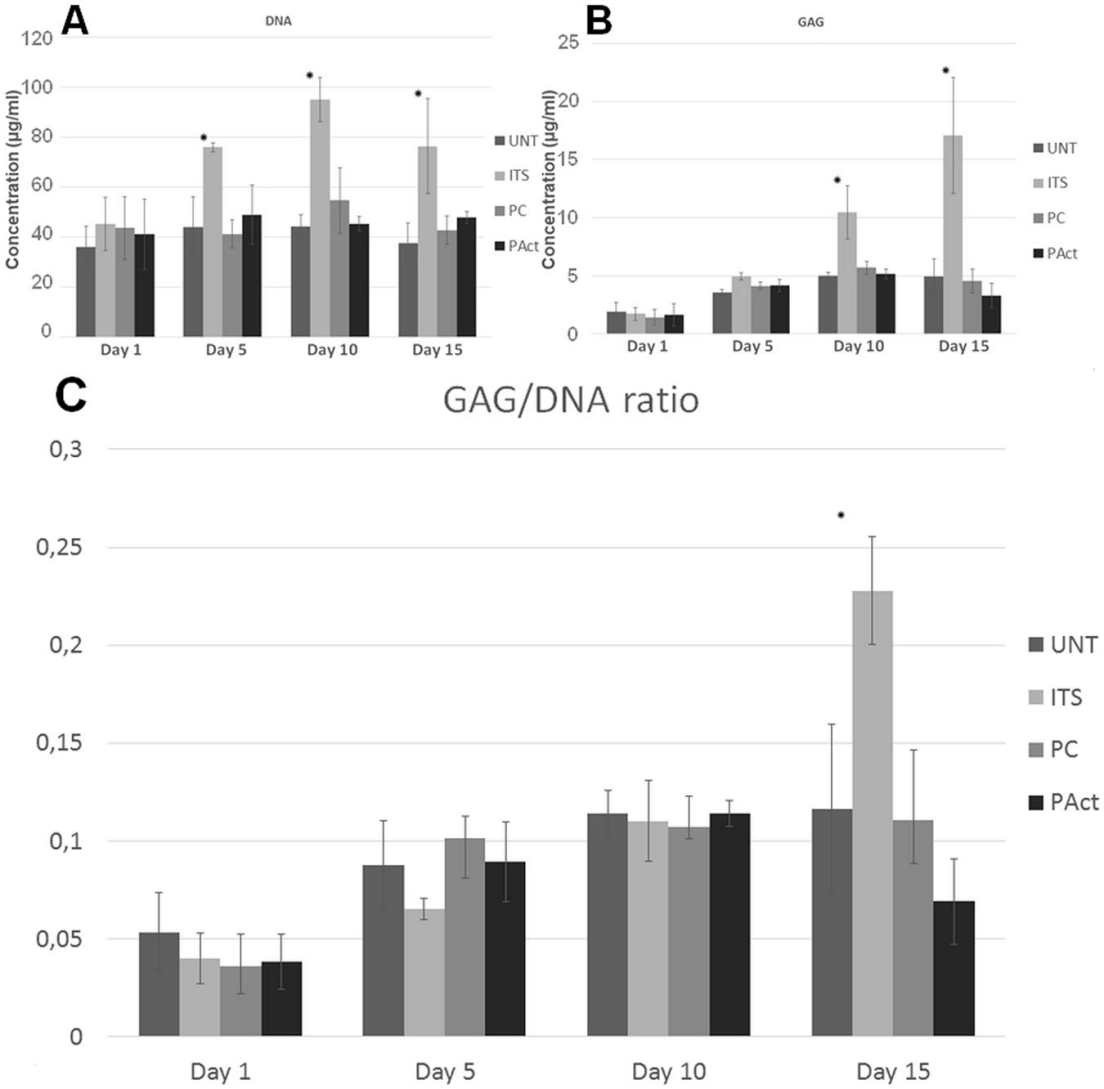
Concentrations of DNA (**A**) and GAG (**B**) and their ratio (**C**) for the first set of experiments determined at fixed time points. *Significantly different compared to the negative control.

The live/dead images of day 1 (Fig. 2A) show remarkable differences in adhesion behavior for the ATDC5 cells. For the negative (UNT) and the positive (ITS) control, the cells exhibited localized clustering, indicating a low affinity towards the scaffold surface. Dark spots, as marked with white arrows in Fig. 2A, were visible for the positive control, indicating cell detachment after staining and hereby confirming the chondroblasts’ low adhesive behavior. The adhesion on plasma treated samples was slightly more homogeneous, with still some signs of clustering visible, probably due to the very high hydrophilicity, something that is not well perceived by chondroblast cell-lines, which usually prefer a more moderate wettability^46,47^. For the plasma coated samples however, a more homogeneous cell spreading is observed, suggesting a higher affinity of the individual cells towards the more moderately hydrophilic scaffold surface compared to the plasma activated sample. According to literature, the altered adhesion behavior of the cells in the first 24 hours after seeding finds its origin in the more efficient adsorption of specific cell adhesion serum proteins such as fibronectin and vitronectin on negatively charged surfaces^48^. Furthermore, it has been proven that carboxylic acid rich surfaces are responsible for the upregulation of focal adhesion components such as vinculin, key factors for an efficient connection between the integrin adhesion molecules and the actin cytoskeleton^24,49,50^. DNA analysis (Fig. 3A) shows that after day 1, the level of DNA for all conditions was similar, suggesting that despite the individual cell behavior, cell seeding efficiency was not significantly influenced by the scaffold surface properties, suggesting that the effects observed by other researchers might be highly cell-dependent.

At day 5 and day 10, based on the live/dead staining, there were no notable differences between the 4 investigated conditions. In all cases, the cells completely covered the individual filaments on the outside, without covering up the pores (Figure SI 2). Based on the DNA data shown in Fig. 3A however, the positive control sample (ITS) showed a significant increase in DNA concentration from day 5 onwards compared to the other samples, a first indication that the plasma activation and coating did not evoke the same reaction on the cells mitotic cycle compared to the ITS soluble growth factors. This initial difference was further confirmed by the live/dead staining at day 15, showing a clear distinction in chondrocyte proliferation between the positive control (ITS) and the other samples (Fig. 2B). The pores of the ITS sample started to close, while the other scaffolds showed no signs of pore filling. The GAG assay (results shown in Fig. 3B) shows that up until day 10 there was no clear distinction between all examined conditions. However, from day 10 onwards, the positive control (ITS) started generating higher amounts of matrix/cell, resulting in a significantly higher GAG/DNA ratio in relation to the other scaffolds as presented in Fig. 3C.

Microscopic cross-section images of the methylene blue stained halved scaffolds were also taken to analyze the cell penetration efficiency throughout the scaffold (Fig. 2C; the reader is advised to view the images online in high resolution)^51,52^. Methylene blue was used to counterstain cells in the scaffolds, showing chondrocytes in dark blue. Albeit characterized with a higher DNA concentration and higher GAG/DNA ratio, there were very little cells found throughout the scaffold for the positive control (ITS). Based on the staining intensity, one could conclude that a sheet of cells is formed around the scaffold rather than throughout the scaffold. This can be considered as non-ideal, given that it closes off the interior of the scaffold from nutrients, inhibiting the proper formation of a 3D cartilage tissue structure. For the other scaffolds, there was a slightly higher penetration efficiency of the chondroblast cells throughout the 3D structure, but the slow mitotic cycle prevented tissue to be formed within a reasonable time period, as the plasma activation and coating did not stimulate the proliferation behavior of the ATDC5 cells.

#### *In vitro* differentiation in ITS enriched medium

Based on these initial results, an identical set of experiments was subsequently conducted, however, this time, ITS soluble growth factors were added to the culture medium (for the samples PAct and PC), using the same concentration as was used for the ITS sample. From this point onwards, the ITS sample acted as the control during this set of experiments and the untreated sample (UNT) is used as a reference towards the first set of experiments.

**Figure 4:**
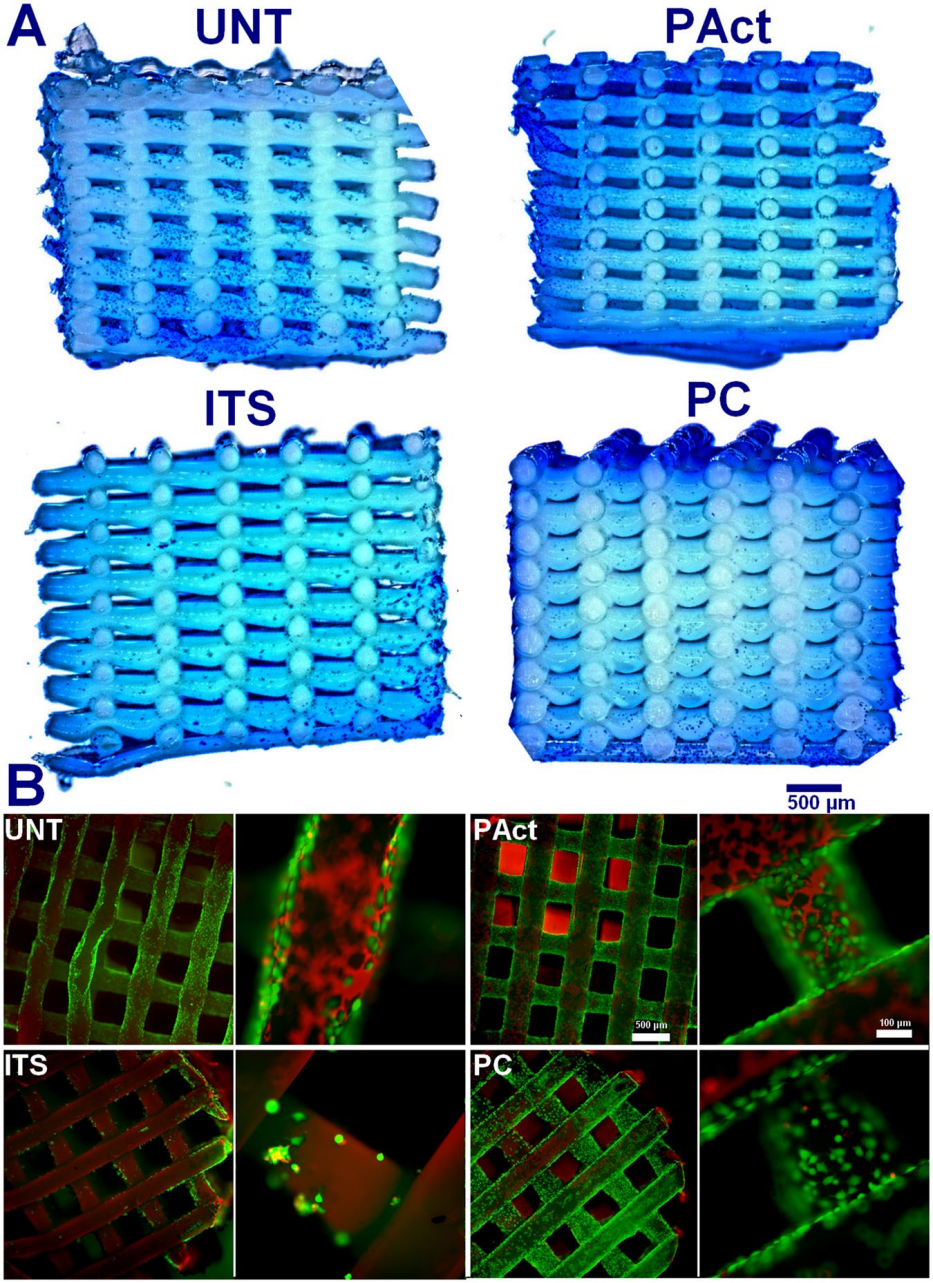
Methylene blue stained cross-sections of the scaffolds for the second set of experiments at day 1 (**A**); live/dead stained images for the second set of experiments at day 1 (**B**).

**Figure 5:**
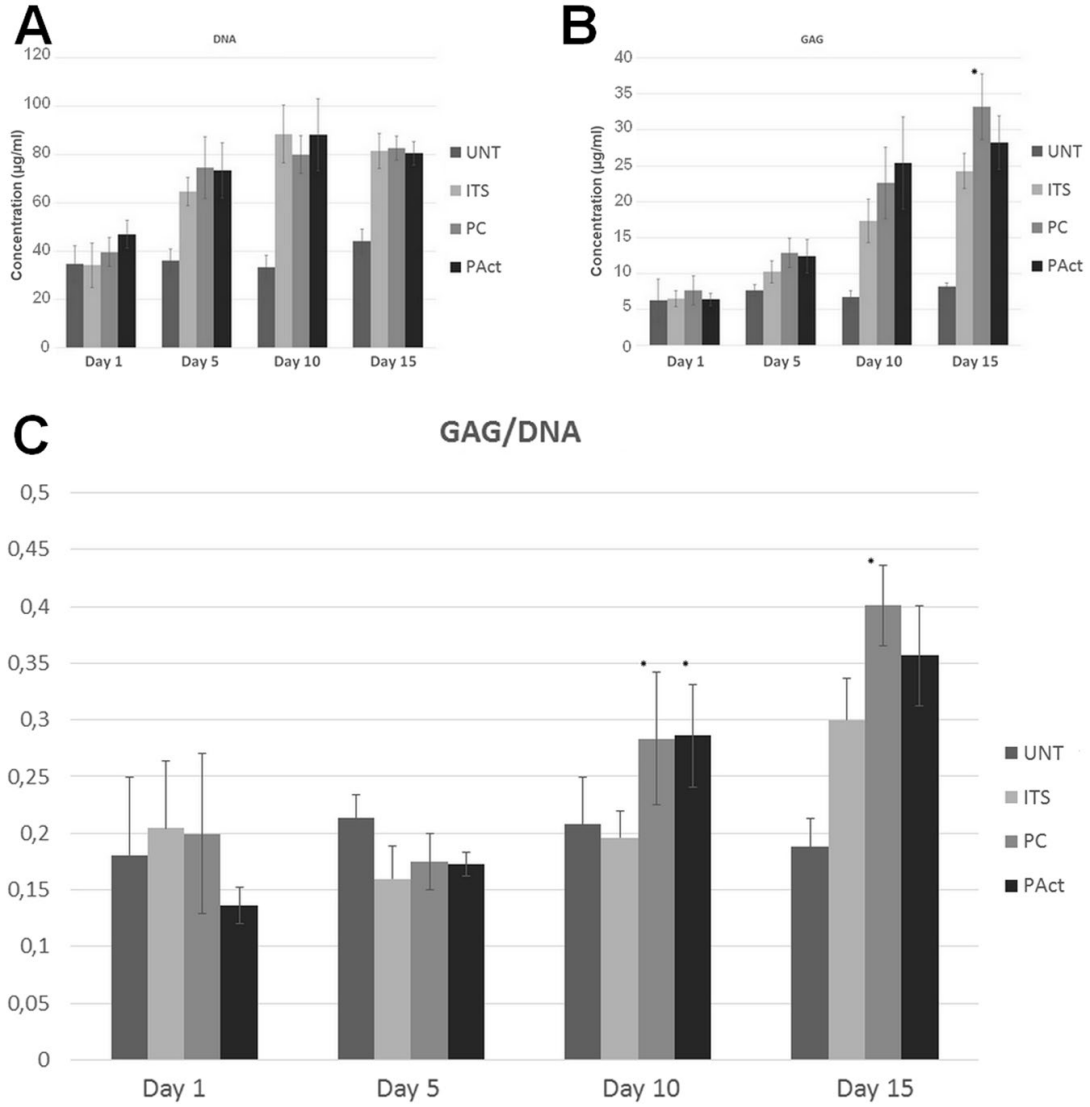
Concentrations of DNA (**A**) and GAG (**B**) and their ratio (**C**) for the second set of experiments determined at fixed time points. *Significantly different compared to the ITS containing sample.

Live/dead staining at time point day 1 (shown in Fig. 4B) revealed similar images compared to the first set of experiments, with the plasma coated sample showing the most homogeneous spread of cells and the highest cell density on top, conclusions which are confirmed by the stereomicroscopic top images of the methylene blue stained scaffolds (presented in Figure SI 4A). Images of cross-sectioned methylene blue stained scaffolds (Fig. 4A) also revealed that the cells were more spread out through the scaffolds UNT and ITS compared to PT and PC, which can be attributed to the higher hydrophilicity of both plasma modified samples (PAct and PC, as shown in Table 1) as the drop of cell suspension was immediately adsorbed within these scaffolds. Combined with gravitational forces, the cells sank in mass to the bottom of the surface modified scaffolds, thus negatively influencing cell distribution. DNA analysis (as shown in Fig. 5) revealed no significant differences at day 1, although the average value seems to be slightly higher on the plasma treated and coated scaffolds (PAct and PC).

**Figure 6:**
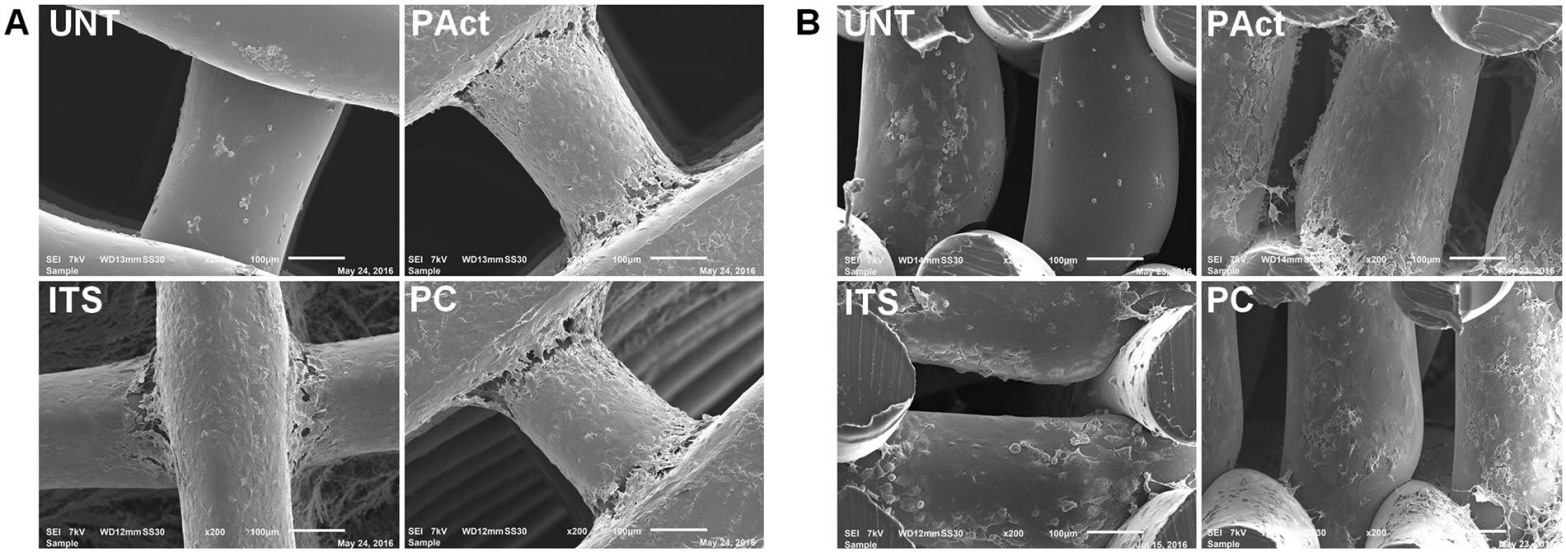
SEM micrographs (top view (**A**) and cross-sectional view (**B**)) for the second set of experiments at day 5.

At day 5, SEM (Fig. 6), live/dead (Figure SI 3A) and methylene blue stained images (Figure SI 4B) showed full coverage of the scaffolds surface filaments with cells for the samples ITS, PAct and PC. For the sample UNT, cell behavior was similar to what was found for the first set of experiments. For all examined samples, the pores were still open, but only the samples incubated in ITS-containing medium (ITS, Pact and PC) showed the first signs of matrix formation in the corners of each pore (Figure SI 3A). Cross-sections of these samples (SEM)) showed some cells throughout the interior of the scaffolds but the production of matrix was limited to the outer surface of the scaffold (Fig. 6).

DNA analysis (Fig. 5A) showed that the addition of the soluble growth factors for the samples PAct and PC significantly stimulated the mitotic cycle, resulting in an increase of DNA by 50–75%, reaching levels similar to the ITS sample. In terms of GAG concentration (Fig. 5B), the samples PC, PAct and ITS showed significantly higher concentrations of GAG compared to the UNT sample, confirming the qualitative live/dead images. The surface-modified scaffolds (PC and PAct) did not show significant differences in GAG levels compared to the ITS samples, yet.

**Figure 7:**
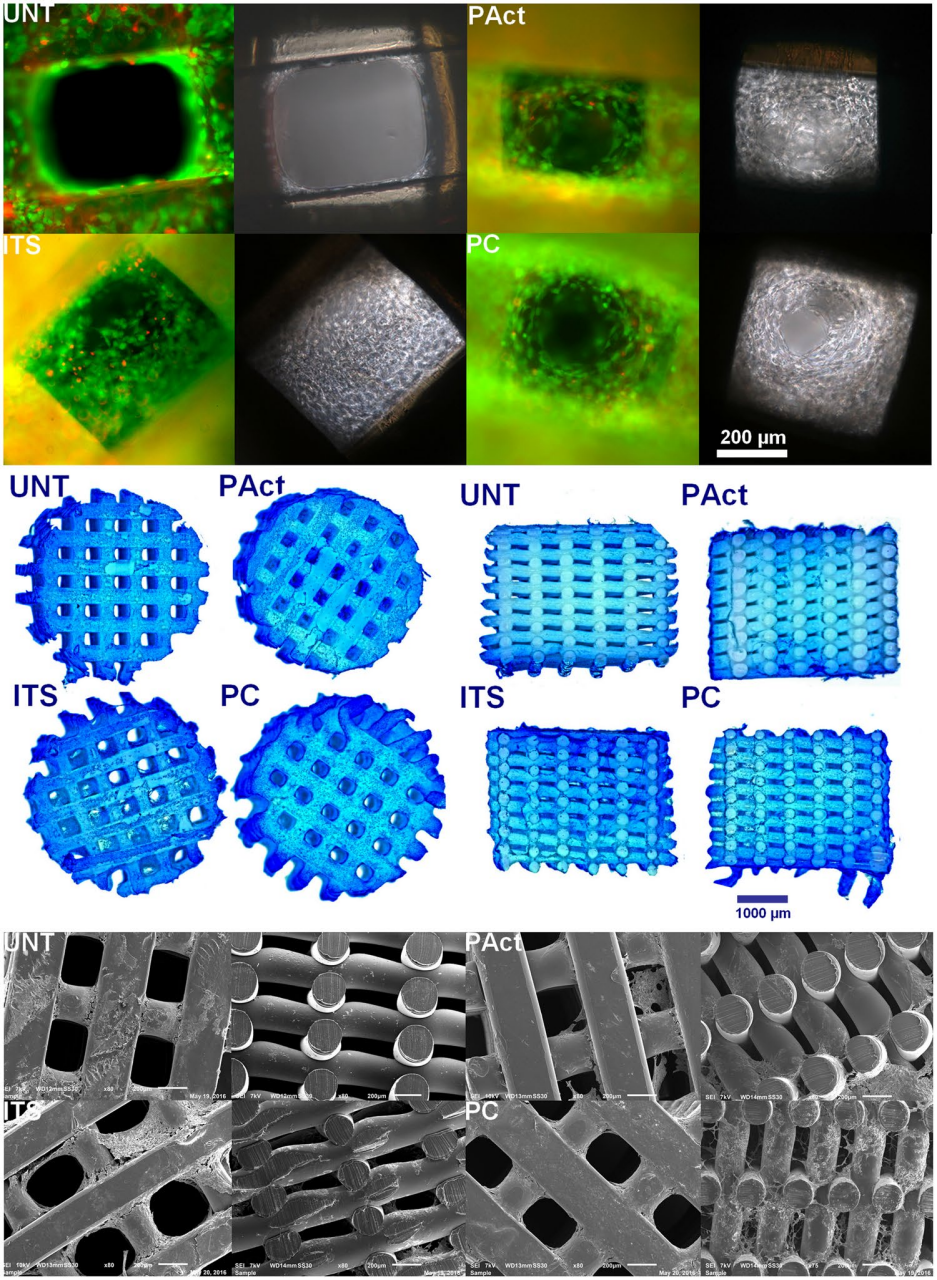
Fluorescent micrographs (left) and bright field (right) images (20x) of live/dead stained samples at time point day 10 (**A**); stereomicroscope images of methylene blue stained scaffolds (left: top view, right: cross-sectional view) at time point day 10 (**B**) and SEM micrographs (80x) (left: top view, right: cross-sectional view) of scaffolds at time point day 10 (**C**).

From day 10 onwards, the differences between the investigated samples became more pronounced. Pores on the outer surface of all scaffolds were (partially) closing up (with the exception of UNT), while the small pores on the side of the scaffolds were already completely closed (Fig. 7A). Cross-sectional analysis however showed that the cells started migrating throughout the scaffolds PC and ITS, while not so much for the sample PAct and not at all for the sample UNT (Fig. 7B). For the plasma coated sample, the cell distribution was homogeneous, while for the ITS sample the distribution of cells and matrix was more clustered. A more localized SEM micrograph analysis showed large portions of the ITS inner surface that were indeed only partially covered by cells, indicating yet again that the affinity of the ATDC5 chondroblasts towards the PEOT/PBT is relatively low (Fig. 7C). For the plasma coated samples however, a dense network of matrix, homogeneously distributed, could be observed in the gaps forming up the porous scaffold (Fig. 7C). For the plasma activated samples, cells were mainly situated on the edges of the scaffold with very little penetration throughout the scaffolds. Again, this phenomenon could most likely be attributed to the inner surface being too hydrophilic. DNA analysis (Fig. 5A) also showed a further increase in DNA concentration for all samples incubated in ITS-rich medium, with no significant differences between them (PC, PAct and ITS). For the GAG analysis (Fig. 5B), the difference between the surface treated samples (PC and PAct) and the ITS sample became more distinctive, but due to a relatively large uncertainty, the difference could not be considered significant. The calculated GAG/DNA ratio (Fig. 5C) however, showed that both the acrylic acid coating as well as the He plasma activation had a significantly positive effect on the formation of GAG matrix. The available literature on the effect of -COOH surface chemistries on chondrogenic differentiation is rather limited, but most papers are in close agreement with the found positive effect the negatively charged surface has on the production of GAGs^53–55^. When expanding the search to similar type of surface modifications (such as hyaluronic acid and chondroitin sulphate) and their effect on chondrogenesis, it becomes evident that it is the presence of a carboxylate anion, specifically, that is responsible for the upregulation of specific genes such as GAG, Aggrecan, SOX9 and collagen II^56–58^.

**Figure 8:**
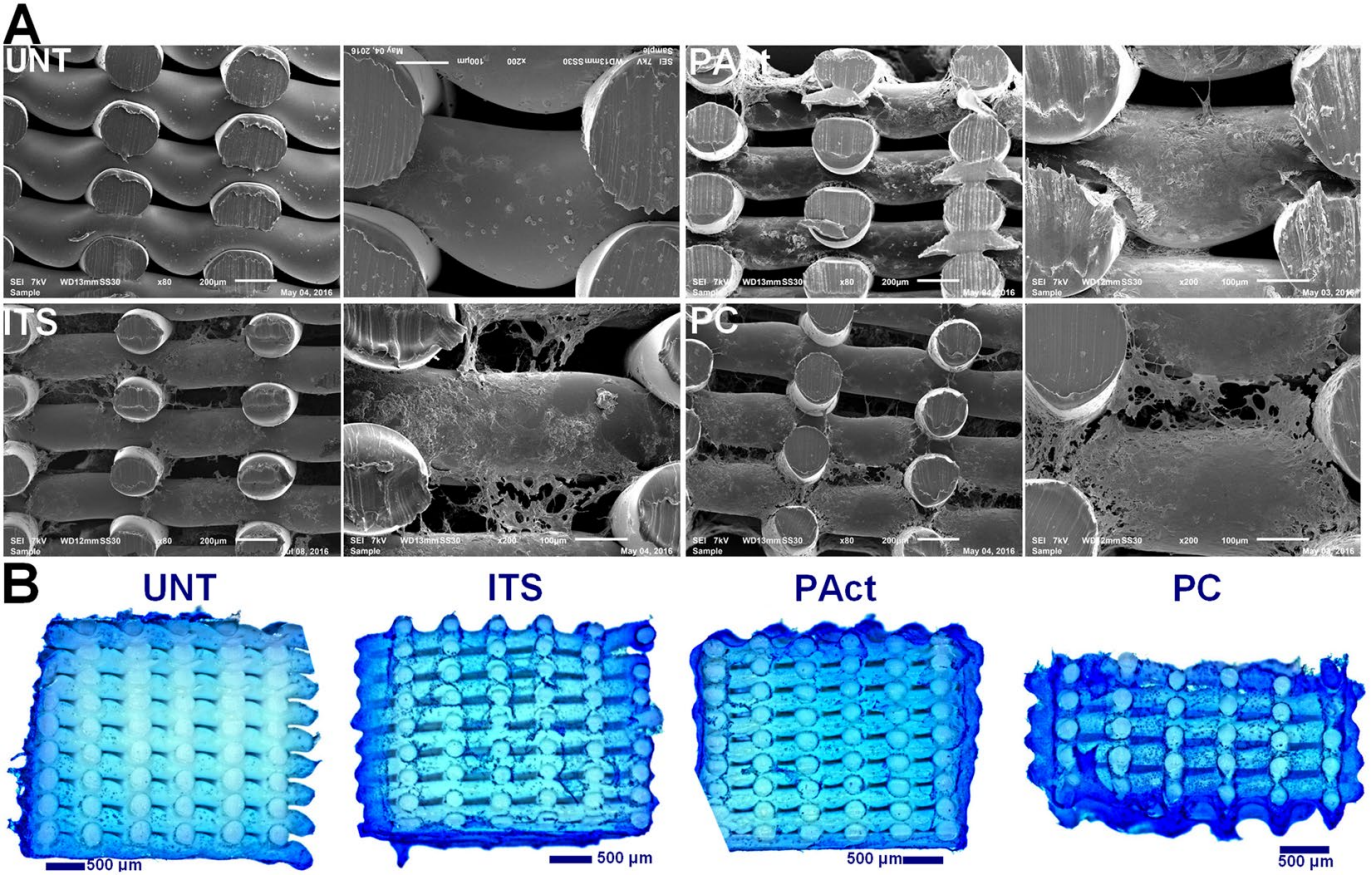
SEM micrographs of cross-sectioned scaffolds (80x and 200x) for the second set of experiments at time point day 15 (**A**) and methylene blue stained cross-sections of the second set of experiments at time point day 15 (**B**).

At day 15, the same trends were observed as for day 10. In the live/dead stained fluorescent micrographs, the sample UNT started showing the first signs of matrix formation, same as what was found at day 5 for the other investigated samples (Figure SI 3A). This confirmed that without boosting the slow mitotic cycle using the soluble growth factors the formation of adult cartilage tissue cannot be achieved within a reasonable time period. This was also quantified by the DNA assay (Fig. 5A), showing a 100% increase in DNA concentration over the 15-day incubation period when ITS was added. For the scaffolds PC, PAct and ITS, no clear distinction could be made based on live/dead images (not included within the paper). Analysis of the cross-sections, using SEM and methylene blue staining (Fig. 8), however showed that the distribution of cells throughout the scaffold differed for each treatment condition, as was the case at day 10. The sample UNT still showed almost no migration of cells throughout the scaffolds, as the majority of cells were situated in the outer regions of the scaffold. The other 3 investigated samples showed the same behavior as described at day 10: the sample PC showed the highest density of cells throughout the scaffold, with matrix formation in between the smaller pores, something that could not be observed at all for the sample PAct and only around the outer edges of the scaffolds of the ITS samples. GAG/DNA quantification showed an even higher difference in GAG/DNA ratio between the samples ITS and PC than what was found at day 10 (Fig. 5C). As the concentrations of DNA remained unchanged (Fig. 5A), it is beyond doubt that the carboxylic acid functional groups were responsible for the significant boost in GAG matrix production. In contrast, the increase in GAG production (Fig. 5B) for the plasma activated sample PAct was not as large as for the plasma coated scaffold and was therefore no longer significantly higher compared to the ITS sample. This lower GAG production rate on the PAct sample could be directly connected with the lower penetration efficiency of the cells into the scaffolds, which in turn could be linked to the higher wettability, as mentioned earlier, as well as the non-specific character of the functional groups introduced by the He plasma activation.

The 2 plasma treatments evaluated in this study induced different cellular mechanisms. In both cases, GAG production was enhanced, which was in accordance with the literature. However, only the plasma coating treatment induced a clear cell migration throughout the 3D scaffolds. Therefore, there remains room for further research on describing how modified surfaces by plasma treatments could have an impact on cellular behavior and specifically the biological molecular events in cells cultured in 3D.

**Figure 9:**
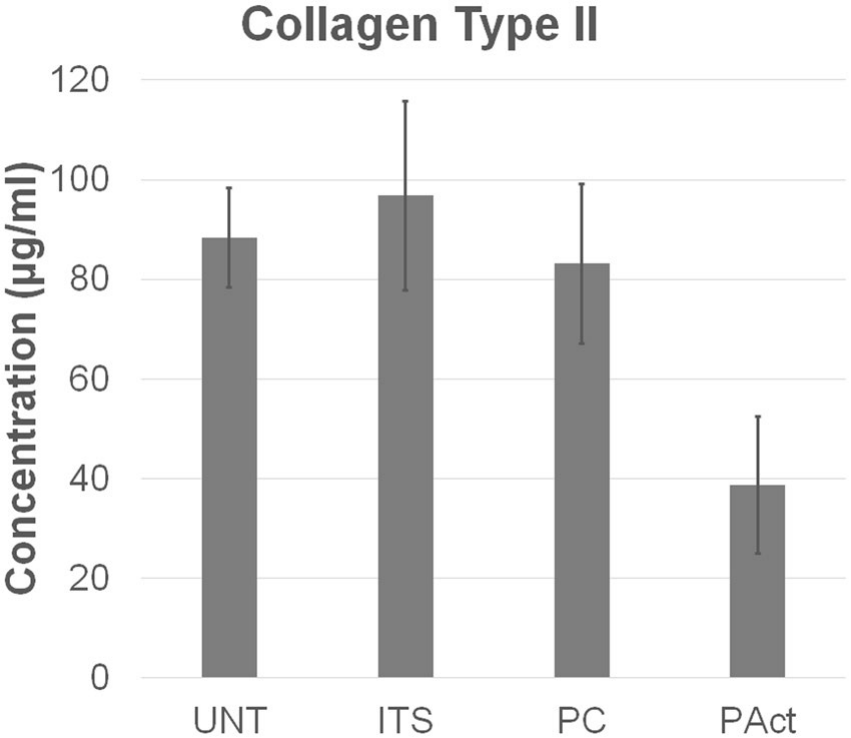
Collagen 2 concentration in the growth medium as determined via ELISA for the second set of experiments at time point day 15.

Collagen 2 ELISA performed on the supernatant of the medium used for incubation during experiment 2 also revealed a significant downregulation of secreted and soluble collagen 2 for the scaffold PAct, something which could not be found for the samples UNT, ITS and PC (Fig. 9). Two possible scenario were taken into account: reduced secretion of collagen 2, or increased nucleation and fibril formation which empty the pool of soluble collagen 2. Although a clear explanation for this cannot be given, the phenomenon could most likely be contributed to functional groups other than carboxylic acid (alcohols, aldehydes, esters) downregulating the secretion of collagen 2 or enhancing fibril formation. Other papers did describe the upregulation of collagen 2 by carboxylic acid rich coatings, which favors the scenario that more collagen 2 fibrils were assembled, reducing the pool of soluble collagen 2 in plasma activated scaffolds^53–55^.

## Conclusion

In this study, PEOT/PBT scaffolds have been fabricated using a 3D additive manufacturing technique resulting in interconnected porous scaffolds with well-defined parameters. Non-thermal plasma technology at medium pressure has subsequently been successfully applied as a surface modification technique for the incorporation of non-specific oxygen containing functional groups (He plasma activation) as well as more specific carboxylic acid functional groups (acrylic acid plasma polymerization). Surface analysis revealed a predominantly chemical surface modification with sufficiently high carboxylic acid incorporation efficiency throughout the scaffold interior to trigger changes in chondroblast behavior all throughout the modified scaffold. The potential of the investigated scaffolds for cartilage tissue engineering has been studied *in vitro* using ATDC5 chondroblasts. The first set of experiments revealed that without the addition of the ITS, the mitotic cycle of the chondroblasts is too slow to achieve a full-grown tissue, the surface modification not having any influence on this. The second set of experiments, where ITS was added, showed that the migration efficiency of the cells through the scaffold is highest for the plasma coated sample, as the plasma activated sample is too hydrophilic and the untreated sample is not supporting sufficient cell-surface interactions. In terms of matrix production, the plasma coated sample was responsible for the highest production of GAG/DNA from 10 days onward. The plasma activation also improved the production of GAG, but, due to its non-specific character, not as efficiently as the plasma coating process. Collagen 2 ELISA revealed some differences between plasma coated and plasma activated samples, but qPCR is required to better understand their origin. Overall, it can be concluded the acrylic acid plasma coated scaffolds have shown potential for cartilage tissue engineering applications.

## Materials and Methods

### Chemicals

He Alphagaz 1 was purchased from Air Liquide and used as such. 300PEOT55PBT45 was received and stored in vacuum to avoid degradation (Polyactive®, PolyVation), (phosphate buffer saline solution) PBS, (Dulbecco’s modified eagle medium) DMEM F12, Fetal bovine serum (FBS) Insulin-Transferrin-Selenium (ITS), Penn/strep, Trypsin, ethidium bromide and calcein-AM were all purchased from Thermo-Fisher Scientific. Acrylic acid (used as such), ethanol 70%, HCl, methylene blue, dimethyl methylene blue (DMMB), chondroitin sulfate, proteinase K, iodoacetamine and Pepstatin A were purchased from Sigma-Aldrich.

### Additive Manufacturing

**Figure 10:**
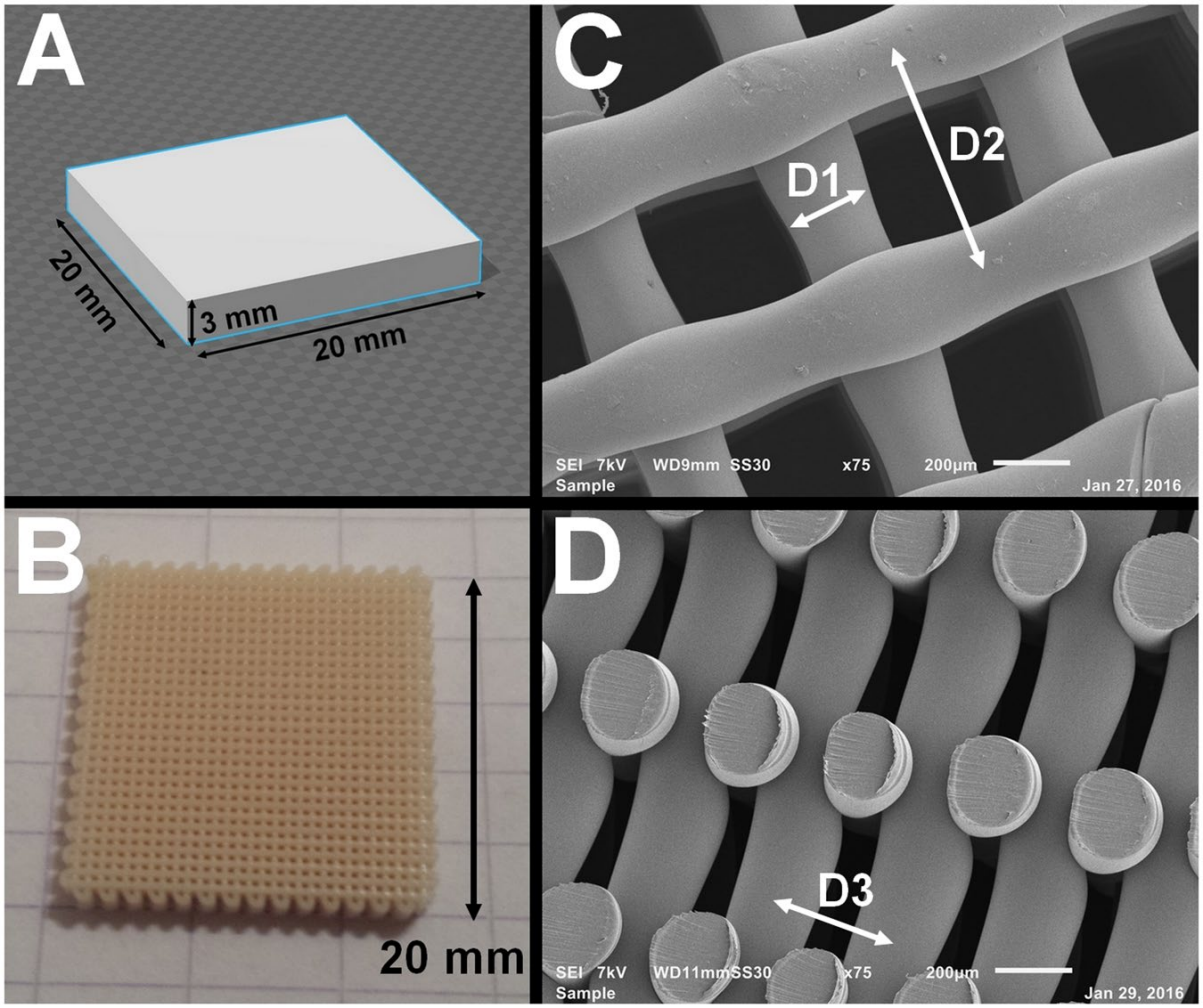
Visualization of the additive manufacturing process: (**A**) Model building in Google Sketch Up; (**B**) Photograph of the printed scaffold (1x magnification); (**C**) Top view SEM micrograph (75x magnification, 200 µm scale bar) showing the filament diameter D1 and the XY filament spacing D2; D) Cross-sectional SEM micrograph (75x magnification, 200 µm scale bar) showing the Z filament spacing.

3D scaffolds were plotted using a commercially available additive manufacturing system (SYS + ENG GMBH, Germany). The PEOT/PBT granules were loaded into a stainless-steel cartridge and heated to a temperature of 195 °C. The cartridge with the molten polymer was then mounted onto the mobile X-Y-Z arm of the printer, where it was encapsulated into a heating element, maintaining the pre-set temperature. A nitrogen pressure of 4.5 bar was applied onto the cartridge to facilitate the printing process, while preventing thermal oxidation. Rectangular blocks (20 × 20 × 3 mm³) were designed using Google Sketch Up^©^ and loaded into the CAM printer software (Fig. 10A). The scaffold was then rendered by the predefined filament diameter, spacing and by the layer thickness, which in turn determined the pore size and overall porosity. The nozzle used to extrude the molten polymer was a stainless-steel precision needle with an internal diameter (ID) of 250 μm and a length of 9.4 mm, purchased from DL technologies. A disposable bio puncher of 4 mm diameter (Integra Milltex) was used to punch out cylindrical samples with a height of 3 mm (UNT). From each printed block (Fig. 10B), 16 cylindrical samples were produced.

### Plasma activation and coating procedure

A parallel-plate DBD reactor was used to perform plasma modifications. The reactor itself has been fully described and characterized in previous work (Figure SI 1)^44^. In short, the cylindrical plasma reactor contains two electrodes placed in parallel 7.7 mm away from each other. The lower electrode is embedded in a ceramic crucible connected to a 50 kHz AC power source (Heinz Bayerle GMBH), while the upper electrode is a porous electrode, covered with a ceramic coating and connected to ground through either a 100 Ohm resistor or a 10.4 nC capacitor. The gas inlet is located at the top of the plasma reactor and the discharge gas first passes through a glass wool filler to distribute the gas flow more evenly before entering the plasma discharge region through the top porous electrode. The bottom of the plasma reactor is connected to a simple pumping unit, allowing the evacuation of the plasma reactor and subsequent filling with a reproducible atmosphere. The plasma activation step (PAct) was performed at 10 kPa in a pure helium atmosphere (gas flow rate of 1 standard liters per minute (slm)), using a discharge power of 2.5 W and a total treatment duration of 2 min. After an exposure of 1 min 45 s, the scaffold was flipped and treated for another 15 seconds in order to efficiently treat all sides of the scaffold. For the plasma polymerization experiments (PC), a helium flow of 7 slm was used in combination with a 0.05 g/h flow of acrylic acid vapor controlled via an El-Flow gas mass flow controller (Bronkhorst) and a µflow liquid mass flow controller (Bronkhorst) respectively. Samples were treated at a discharge power of 18 W and a pressure of 50 kPa for a total time of 10 min, being flipped after 5 min in an effort to efficiently and homogeneously deposit the coating inside the scaffold. Before performing the plasma polymerization step, the previously described plasma activation step was also performed in order to increase adhesion between the scaffold and the initial plasma polymer layer.

### Scaffold characterization

#### Scaffold geometry

Scaffold geometry was characterized with scanning electron microscopy (SEM) (JEOL 6000). Before the analysis, the samples were coated with gold, using a gold sputter coater (JFC-1300 autofine coater, JEOL, Japan). Measurements were done at an accelerating voltage of 7 kV and spot size 30 and micrographs were recorded at 80x −200x and 500x magnifications. while porosity was calculated according to the formulas provided by Moroni *et al*.^16^:

1 $$P=1-\frac{{V}_{scaffold}}{{V}_{cube}}=1-\frac{\pi }{4}\,\ast \,\frac{1}{\frac{D2}{D1}}\,\ast \,\frac{1}{\frac{D3}{D1}}$$

where P is the porosity, D1 the diameter of the printed filament, D2 the filament XY spacing and D3 the Z spacing, as is shown in Fig. 10C and D.

#### Scaffold topography

Changes in scaffold topography were analyzed on a microscale with SEM (JEOL 6000) and on a nanoscale with atomic force microscopy (AFM) (Park XE-70). For the AFM micrographs, scans of 15 µm² were recorded in non-contact mode with a silicon cantilever (Nanosensors^TM^, PPP-NCHR) and XEP software was used for surface roughness analysis after an X and Y plane auto-fit procedure was applied to the recorded images. For each condition, three different Regions of interest on a single sample were examined.

#### Wettability and surface chemical composition

Changes in surface wettability induced by the plasma activation and polymerization methods have been measured on spin-coated PEOT/PBT cover slips (2% w/w PEOT/PBT in chloroform, 2000 rpm). A KRÜSS Easy Drop system was used to measure the static water contact angle (WCA) values. Droplets of 1 µl were deposited onto the surface, after which a Laplace-Young fitting was applied. For each sample, the water contact angle was determined on 6 different locations, from which an average value and standard deviation were calculated.

The surface chemical composition throughout the scaffold was analyzed using XPS (PHI 5000 Versaprobe II) employing an Al K_α_ X-ray source (hν = 1486.6 eV) operated at 50 W. All measurements were conducted in a vacuum of at least 10^−6^ Pa and the photoelectrons were detected with a hemispherical analyzer positioned at an angle of 45° with respect to the normal of the sample surface. Survey scans and individual high resolution C1s spectra were recorded with a pass energy of 187.85 eV and 23.5 eV respectively. The elemental composition was determined from the survey scans and quantified with Multipak software (V9.6.1) using a Shirley background and applying the relative sensitivity factors supplied by the manufacturer of the instrument. Multipak software was also applied to curve fit the high resolution C1s peaks after the hydrocarbon component of each C1s spectrum (285.0 eV) was used to calibrate the energy scale. In a next step, the peaks were deconvoluted using Gaussian–Lorentzian peak shapes and the full-width at half maximum (FWHM) of each line shape was constrained below 1.5 eV.

### Cell expansion and seeding

ATDC5 cells (Passage 10–14) were seeded with a density of 6500 cells/cm² in 175 T-flasks. DMEM F-12 with 5% FBS and 1% Penn/strep was used as culture medium. For the ITS-containing medium 10 µl/ml ITS was added. T-flasks were incubated at 37 °C with 5% CO_2_. Doubling time of the cells is approximately 16 h, resulting in the re-expansion of the cells after 3 days. Trypsin was used (3 ml) to detach the cells, after which they were suspended in culture medium. 35 µl of suspension, containing 500 000 cells was pipetted on top of the scaffold. After 4 h, 1.5 ml of culture medium was added to the 24-well plate containing the scaffolds. Cells were incubated at 37 °C with 5% of CO_2_. After 24 h, the scaffolds were put in fresh plates to discard unattached cells present at the bottom of the plates. The medium was refreshed every 2 days.

Upon the predefined time points, the scaffolds were removed from the medium and either stained for fluorescence and stereomicroscopy, or dried, quartered and stored at −80° for the quantitative analyses of GAG and DNA. For the SEM and Methylene blue stainings, scaffolds were immersed in a paraformaldehyde solution for 30 min to fixate the cells. Samples were then stored at 4 °C. Culture medium was also stored at −80 °C for the quantitative analysis of collagen 2.

### Viability assay

Analysis of cell viability was done via live/dead staining. After washing the scaffolds 3 times with PBS, calcein AM (1:12500) and ethidium bromide (1:4000) were dissolved in PBS and added to the scaffolds (500 µl). After 25 min incubation at 37 °C (dark), the scaffolds were washed with PBS and stored in a dark container. A Nikon Ti-S Inverse Fluorescence microscope equipped with a Nikon DS-Ri2 camera was used to evaluate cell viability (live, λ = 498 nm)/(dead, λ = 590 nm) images (4x and 20x) of the scaffolds.

### Methylene blue staining

Methylene blue (0.05%) was dissolved in distilled water. Scaffolds were incubated for 30 seconds in the solution, and washed 5 times with water, after which they were immediately imaged using a Nikon Ti-S stereomicroscope.

### GAG/DNA assay

After thawing, the quartered scaffolds were lysated for 16 h at 56 °C with a Tris/EDTA buffer containing 1 mg/mL proteinase K, 185 μg/mL iodoacetamine and 10 μg/mL Pepstatin A. The quantification of the total amount of DNA was performed using a CyQuant^®^ DNA assay (Molecular Probes, Eugene, USA) as previously reported^59^. Lysate was pipetted in duplicate to a black 96-well plate, followed by addition of NaCl-EDTA buffer containing component B of the kit (20x) and RNase (1000x). The plate was incubated for 1 h at room temperature. Subsequently, Gr-dye solution was added and the samples were incubated for 15 min. The fluorescence signal was determined using a spectrophotometer (CLARIOstar microplate reader, BMG Labtech) at an excitation wavelength of 480 nm and emission wavelength of 520 nm.

The GAG levels were spectrophotometrically determined after reaction with 16 mg of DMMB in a 10 mM hydrochloric acid solution containing 3.04 g/L of glycine and 2.37 g/L of NaCl. The absorbance was measured on a micro plate reader (ClARIOstar microplate reader, BMG Labtech). The amount of GAG was calculated using a standard of chondroitin sulfate (Sigma–Aldrich), as reported by Chen *et al*.^60^.

### Collagen 2 assay

A collagen type 2 ELISA kit was bought from Abexxa (46.88–3000 pg/ml) and used according to the manual of the manufacturer. Scaffold Incubation medium for all conditions was collected at day 15 and stored at −80 °C. After thawing, the medium was centrifuged at 300 G for 5 minutes to precipitate all solids present in the medium. Each condition was analyzed in triplicate (CLARIOstar microplate reader, BMG Labtech) at an excitation wavelength of 480 nm and emission wavelength of 520 nm

### Statistical analysis

Results were averaged and standard deviations were calculated, followed by an ANOVA analysis in combination with a Tukey’s post-hoc test. if the p-value was below 0.05, results were considered to be significantly different. For AFM, 3 samples were analyzed with 3 random points per sample. For XPS 3 samples were measured, with single points defined in an XY array as can be seen in Fig. 10. For WCA 3 samples were measured, with 6 points per sample. For Live/dead staining, methylene blue and SEM, a single sample was used per time point per condition. For GAG, DNA and collagen 2 analyses, 5 samples were analyzed per condition per time point.

## Electronic supplementary material

Supplementary information
