# Supplementary material for: Acrylic Acid Plasma Coated 3D Scaffolds for Cartilage tissue engineering applications
Source: Sci Rep. 2018 Mar 1;8:3830. doi: 10.1038/s41598-018-22301-0 (PMC5832775; doi:10.1038/s41598-018-22301-0)
Supplement: Supplementary file 1 — Supplementary information [file 41598_2018_22301_MOESM1_ESM.pdf]

Acrylic Acid Plasma Coated 3D Scaffolds for Cartilage tissue engineering applications

Pieter Cools[1], Carlos Mota[2], Ivan Lorenzo-Moldero[2], Rouba Ghobeira[1], Nathalie De Geyter[1], Lorenzo Moroni[2*], Rino Morent[1*]

[1]Research Unit Plasma Technology, Department of Applied Physics, Sint-Pietersnieuwstraat 41 B4, Ghent University, 9000 Ghent - Belgium
[2]Department of Complex Tissue Regeneration, MERLN Institute for Technology Inspired Regenerative Medicine, Universiteitssingel 40, University of Maastricht, 6200 MD, Maastricht, The Netherlands

e-mail addresses corresponding authors: L.Moroni@MaastrichtUniversity.nl; Rino.Morent@Ugent.be

Supporting information

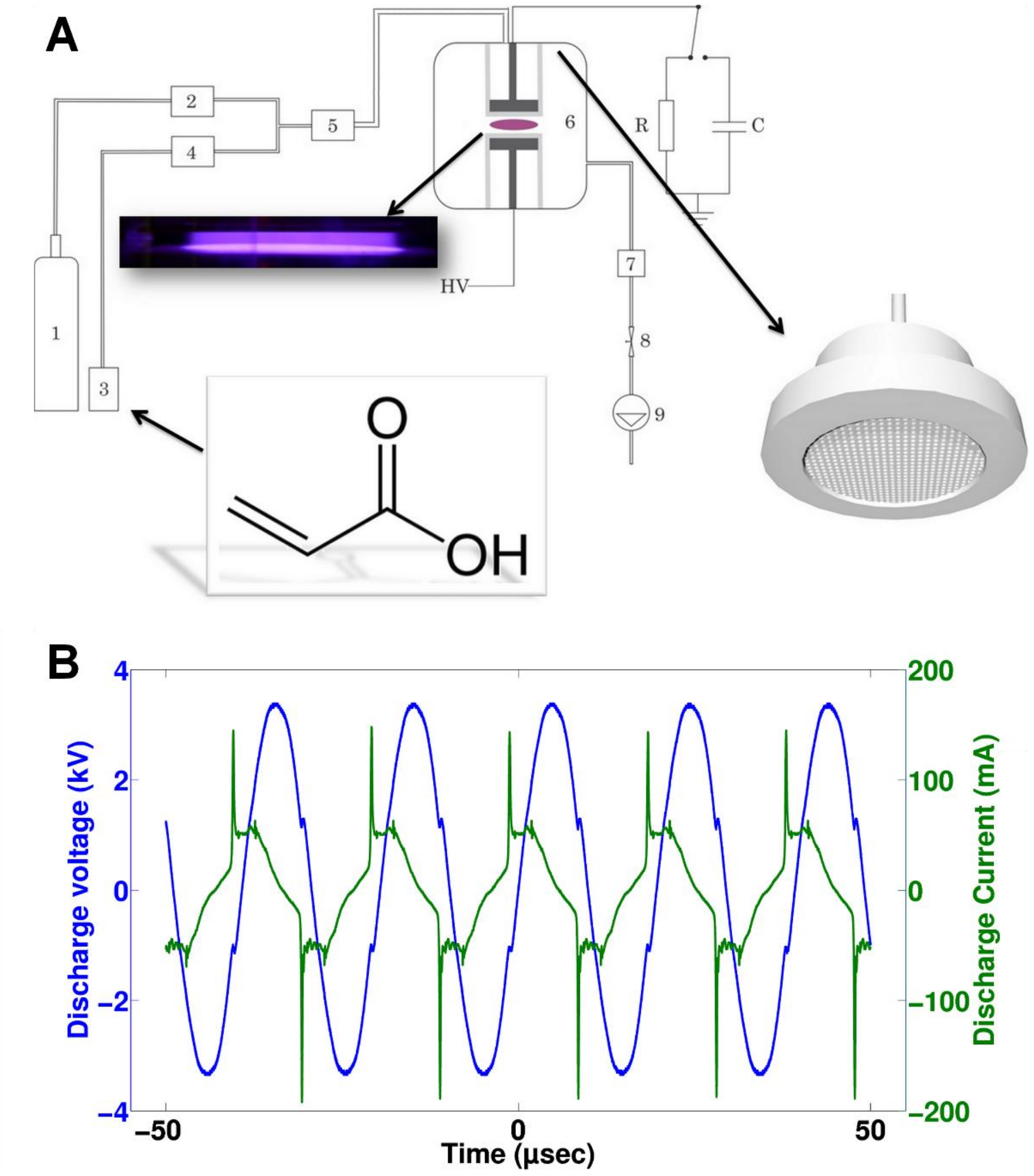

Figure SI1. Schematic representation of the non-thermal plasma reactor: 1) Helium bottle; 2) Helium gas flow controller; 3) Container filled with acrylic acid; 4) Acrylic acid liquid flow controller; 5) Controlled evaporation and mixing system allowing for a homogeneous gas flow containing acrylic acid vapor; 6) Plasma reactor with a porous top electrode; 7) Manometer; 8) Closing valve to control pressure inside chamber; 9) Pumping unit[44] (reproduced with permission from Cools, P. *et al.* Influence of DBD Inlet Geometry on the Homogeneity of Plasma-Polymerized Acrylic Acid Films: The Use of a Microplasma–

Electrode Inlet Configuration. *Plasma Process. Polym.* **12**, 1153-1163, doi:10.1002/ppap.201500007 (2015)). B) voltage-current waveplot of the plasma discharge during acrylic acid polymerization

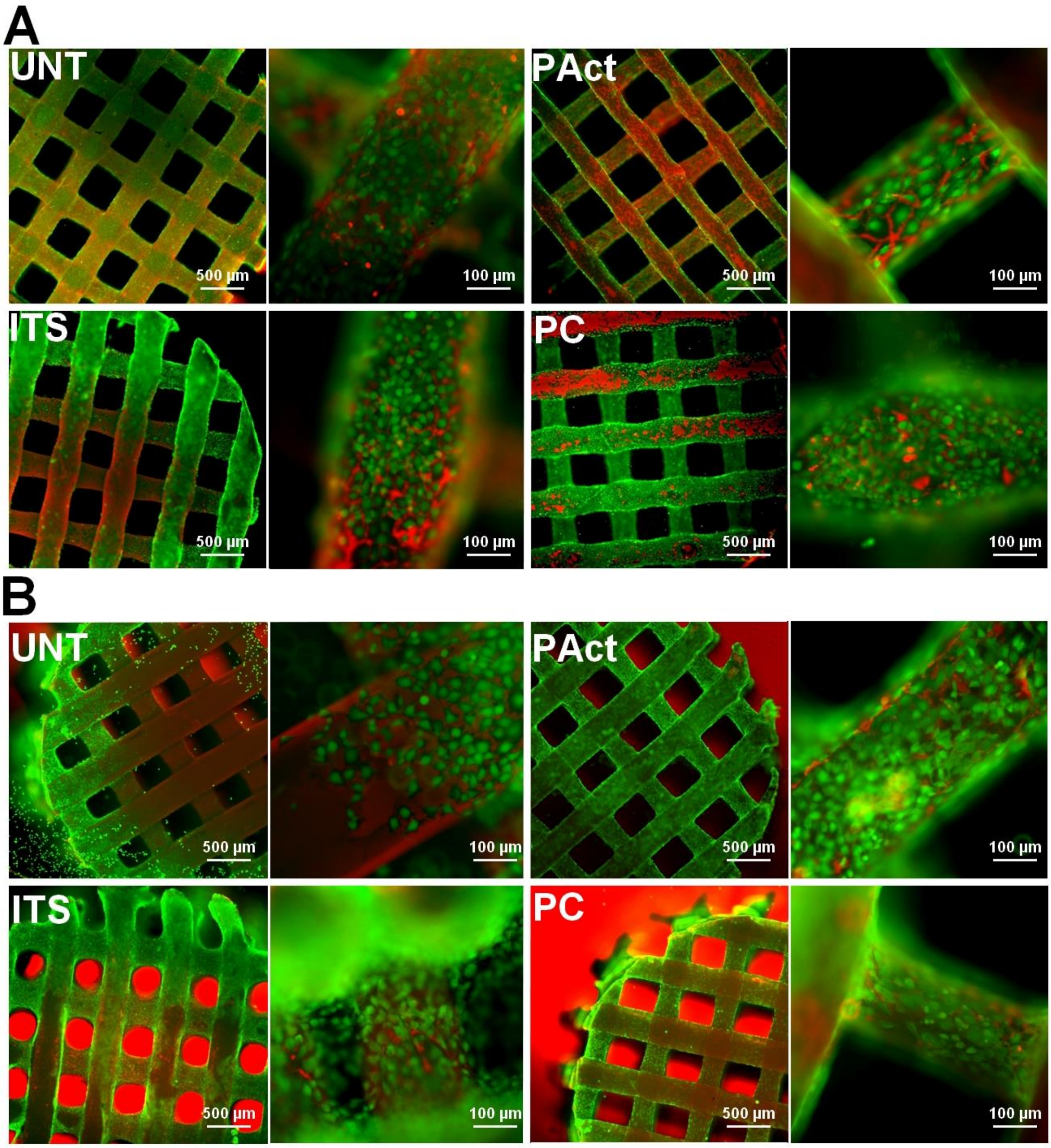

Figure SI2. Fluorescent micrographs (4x and 20x) of live/dead stained samples for the first set of experiments at time points A) day 5 day and B) day 10.

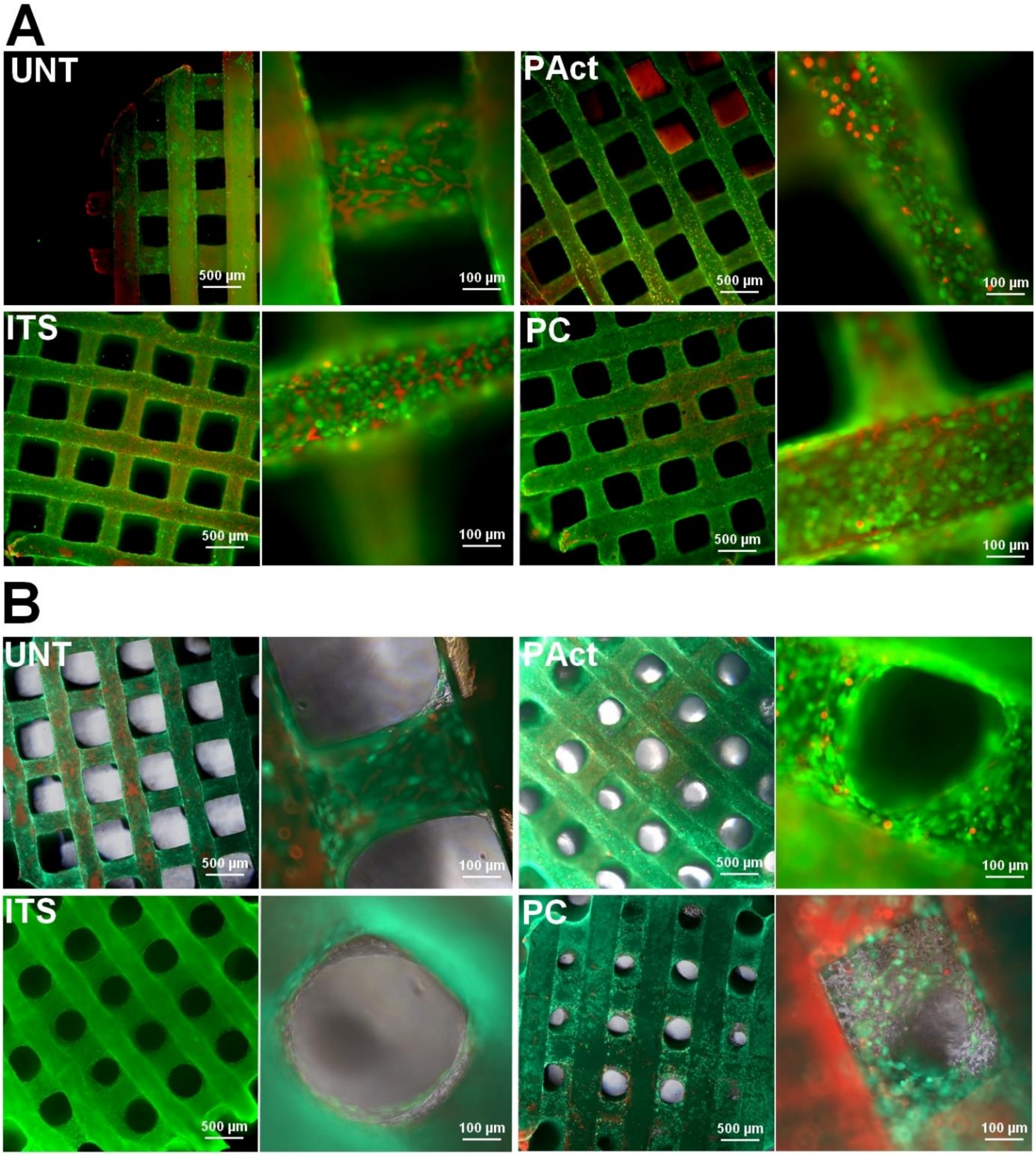

Figure SI3: Fluorescent micrographs (4x and 20x) of live/dead stained samples for the second series of experiments at time points A) day 5 and B) day 10.

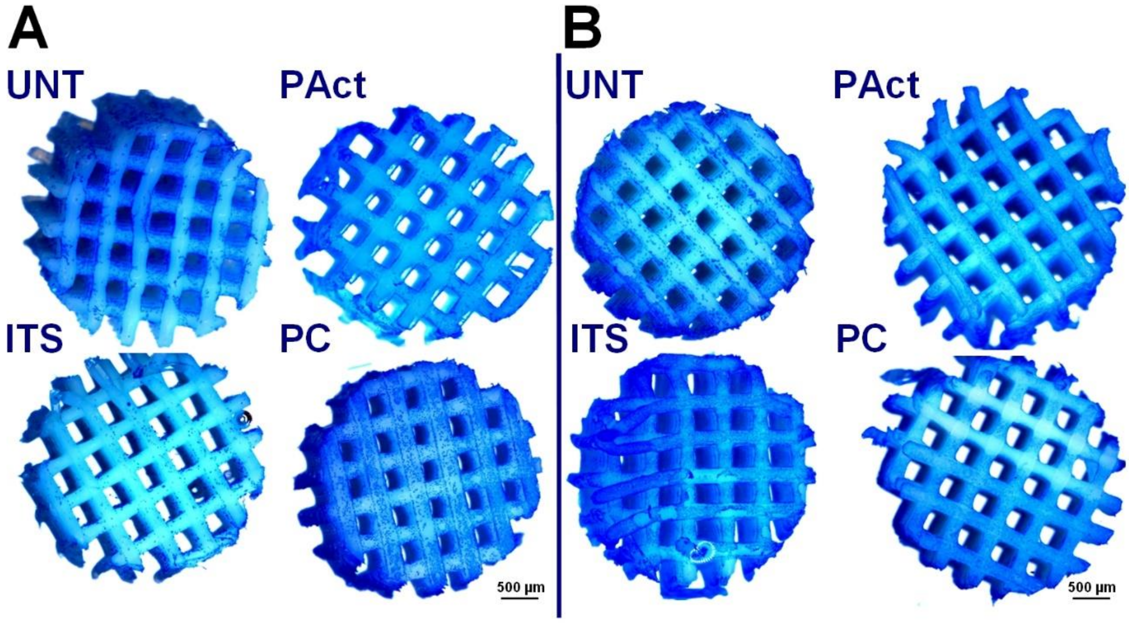

Figure SI4. Stereomicroscope top images of methylene blue stained scaffolds for the second set of experiments after A) 1 day and B) 5 days.
